# Non-Hodgkin lymphoma with relapses in the lacrimal glands

**DOI:** 10.3205/oc000026

**Published:** 2015-06-03

**Authors:** Rita Couceiro, Helena Proença, Filomena Pinto, Ana Fonseca, Manuel Monteiro-Grillo

**Affiliations:** 1 Hospital de Santa Maria, Department of Ophthalmology, Lisbon, Portugal

**Keywords:** lacrimal gland, non-Hodgkin lymphoma relapse, ocular ultrasound, orbit

## Abstract

**Objective:** To report an unusual case of systemic non-Hodgkin lymphoma (NHL) with repeated relapse in the lacrimal glands, in spite of complete remission for several years after treatment.

**Methods:** A 78-year-old male with small lymphocytic B cell NHL, stage IV disease (lung invasion), was submitted to surgery and chemotherapy in 2001, with complete remission of the disease. In 2003 he developed a nodular lesion in the right lacrimal fossa. Pathology results revealed a local relapse of NHL. Radiation and chemotherapy were initiated and complete remission was again achieved. In 2012 the patient developed a new nodular lesion located in the left lacrimal fossa, resulting in diplopia, ptosis and proptosis of the left eye. Orbital computerized tomography (CT), ocular ultrasound and incisional biopsy were performed.

**Results:** Orbital CT revealed a lesion infiltrating the left lacrimal gland and encircling the globe. Biopsy results confirmed a local relapse of B cell NHL. The patient was submitted to local radiation therapy with progressive resolution of ptosis, proptosis and diplopia. Response to treatment was monitored with ocular ultrasound.

**Conclusions:** Patients with NHL diagnosis should be immediately investigated if ophthalmic or orbital symptoms develop. NHL extension to the orbit and adnexa is infrequent (5% of NHL cases) but may occur at any stage of the disease, including as a relapse site. In such cases, radiation and chemotherapy achieve good results, inducing long periods of remission.

## Introduction

Systemic non-Hodgkin’s lymphoma (NHL) is reported to have secondary extension to the orbit and adnexa in about 5% of cases [1] and an orbital presentation in 2.4% of all NHL cases [2]. However, given that clinical manifestations of orbital lymphoma are sometimes subtle, some authors suggest that this prevalence might be higher [1]. Patients with NHL diagnosis should therefore be suspected of having orbital involvement if an orbital mass, ptosis, proptosis or lid edema develop [1]. On the other hand, patients with primary orbital and ocular adnexal lymphoma evidence extraorbital involvement in more than 50% of cases, which determines the need for extensive systemic workup at diagnosis [3].

The typical presentation of adnexal lymphoproliferative disease includes a painless mass, swelling or proptosis [4]. Involvement of adnexal structures in systemic NHL may appear at any time during the course of the disease, including as a relapse site [1], [5].

Treatment with systemic chemotherapy or systemic immunotherapy and combination with local radiation treatment is beneficial in secondary orbital lymphomas, inducing prolonged remission [1], [6].

With this report we aim to describe a rare case of systemic non-Hodgkin lymphoma (NHL), with repeated relapses in the lacrimal glands, in spite of complete and sustained remission after treatment.

## Case description

A 78-year-old caucasian male, without any prior relevant medical history, was diagnosed with small lymphocytic B cell NHL in 2000, presenting with stage IV disease with lung invasion. He was submitted to surgery and chemotherapy (cyclophosphamide, vincristine, prednisone; 8 cycles), with complete regression of the disease. 

In 2003 the patient developed intermittent diplopia in right gaze and a nodular lesion was noted at the lateral portion of his right orbit. CT imaging revealed a lesion in the right lacrimal fossa, pressing the globe down and forward without invading it. An incisional biopsy was performed and pathology results revealed local recurrence of NHL, but no immunohistochemistry analysis was conducted due to a small sample size with artifactual changes. The patient underwent chemotherapy (cyclophosphamide, hydroxydaunorubicin, oncovin, prednisone; 4 cycles) and radiation therapy (12 cycles), with complete and sustained response to treatment.

In 2012 the patient developed progressive ptosis of the left eye (OS) and intermittent diplopia, with no other visual complaints. Concurrently, a new nodular lesion was noted in the lateral portion of the left orbit.

On observation the patient presented best corrected visual acuities (BCVA) of 20/25 in both eyes. Inferior dystopia and proptosis in OS were noted; Hertel examination (106 mm) results were 15 mm in the right eye and 20 mm in OS. There was slight restriction of movements in OS in up and left gaze, resulting in diplopia. A clear enlargement of the left lacrimal gland was evident on examination, with discrete associated conjunctival chemosis. The remaining anterior segment features and fundoscopy were unremarkable.

**Figure 1:**
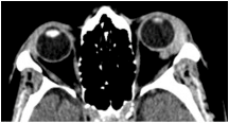
Orbital CT, axial view shows an infiltrative lesion, located in the left lacrimal gland.

Orbital CT imaging revealed an infiltrative hyperdense lesion in the left lacrimal gland, encircling the globe, the muscle cone and the lateral and proximal portions of the optic nerve, without invading any of these structures, nor the extraocular muscles or the orbital walls (Figure 1 (Fig. 1)). On ocular ultrasound the lacrimal gland lesion exhibited well defined borders, internal homogeneous structure and medium/low reflectivity. 

Incisional biopsy of the left lacrimal gland was performed and pathology results revealed B cell NHL relapse. Immunohistochemistry results were positive for CD20 and bcl-2 and negative for CD5, CD10, CD23, CD43 and cyclin D1. 

**Figure 2:**
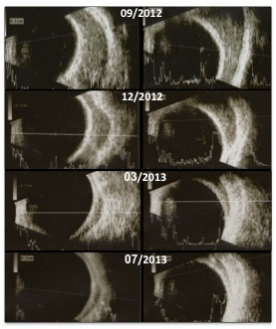
Consecutive ultrasounds of the left eye – superior transversal view (left column) and lateral transversal view (right column) – documenting progressive resolution of the lacrimal gland lesion after radiation therapy treatment was performed in November 2012.

Local radiation therapy (total dosing: 8 Gy/2 fractions) was undertaken and during the following 6 months there was a gradual resolution of diplopia, ptosis and proptosis in OS. Progressive reduction in lesion size was monitored with ultrasound until its complete regression (Figure 2 (Fig. 2)). At last follow-up BCVA remained 20/25 bilaterally.

## Discussion

The differential diagnosis of an orbital mass lesion is vast [7] and although the orbit is rarely a secondary site of lymphoma dissemination, patients with NHL diagnosis should be immediately investigated if ophthalmic or orbital symptoms develop [1]. 

The reported case is unusual for the repeated NHL relapses in the lacrimal glands, separated by several years of complete remission. Orbit and adnexa NHL have indeed been documented in relapses of previously diagnosed lymphomas [5]. 

In the reported case, the patient was initially diagnosed with small lymphocytic B cell NHL, which is considered an indolent lymphoma with a variable clinical course [8]. This might include relapses at the original site or at new locations and even a change in lymphoma histology can occur [9]. Immunohistochemistry analysis was not performed in the first relapse, but in 2012 results suggest the existence of a MALT subtype NHL (CD20+, CD5–, CD10–, CD23–), confirming a change in NHL histology. However, this does not represent a change into a more aggressive histology and therefore, we believe, should not influence the patient’s survival. 

Radiation and chemotherapy have proved to achieve good results in secondary orbital NHL [1], [6] and were effective in treating both relapses in this case. Sustained remission after relapse of indolent lymphomas can often be obtained as long as the disease histology remains low-grade, although relapse will usually ensue [9], determining the need for prolonged follow-up.

## Conclusions

Patients with NHL diagnosis should be immediately investigated if ophthalmic or orbital symptoms develop. NHL extension to the orbit and adnexa is infrequent (5% of NHL cases) but may occur at any stage of the disease, including as a relapse site. In such cases, radiation and chemotherapy achieve good results, inducing long periods of remission.

## Notes

### Competing interests

The authors declare that they have no competing interests. No financial support was received for this submission.

### Informed consent

This case report was performed with informed consent regarding the consultation of human medical records. 

### Acknowledgments

Dr. Ana Cristina Ferreira, Dr. Dolores López-Presa – Department of Pathology, Hospital de Santa Maria, Lisbon, Portugal.
